# Antibiotic Treatment Shapes the Antigenic Environment During Chronic TB Infection, Offering Novel Targets for Therapeutic Vaccination

**DOI:** 10.3389/fimmu.2020.00680

**Published:** 2020-04-28

**Authors:** Yu-Min Chuang, Noton K. Dutta, James T. Gordy, Victoria L. Campodónico, Michael L. Pinn, Richard B. Markham, Chien-Fu Hung, Petros C. Karakousis

**Affiliations:** ^1^Department of Pathology, Johns Hopkins University School of Medicine, Baltimore, MD, United States; ^2^Department of Medicine, Johns Hopkins University School of Medicine, Baltimore, MD, United States; ^3^W. Harry Feinstone Department of Molecular Microbiology and Immunology, Johns Hopkins Bloomberg School of Public Health, Baltimore, MD, United States; ^4^Department of International Health, Johns Hopkins Bloomberg School of Public Health, Baltimore, MD, United States

**Keywords:** *Mycobacterium tuberculosis*, tuberculosis DNA vaccines, persistence, stringent response, immunotherapy

## Abstract

The lengthy and complicated current regimen required to treat drug-susceptible tuberculosis (TB) reflects the ability of *Mycobacterium tuberculosis* (Mtb) to persist in host tissues. The stringent response pathway, governed by the dual (p)ppGpp synthetase/hydrolase, Rel_*Mtb*_, is a major mechanism underlying Mtb persistence and antibiotic tolerance. In the current study, we addressed the hypothesis that Rel_*Mtb*_ is a “persistence antigen” presented during TB chemotherapy and that enhanced immunity to Rel_*Mtb*_ can enhance the tuberculocidal activity of the first-line anti-TB drug, isoniazid, which has reduced efficacy against Mtb persisters. C57BL/6 mice and Hartley guinea pigs were aerosol-infected with *M. tuberculosis* (Mtb) and, 4 weeks later, received either human-equivalent daily doses of isoniazid alone, or isoniazid in combination with a DNA vaccine targeting *rel*_*Mtb*_. After isoniazid treatment, there was a significant reduction in dominant antigen ESAT6-reactive CD4^+^ or TB10.4-reactive CD8^+^ T cells in the lungs and spleens of mice. However, the total number of Rel_*Mtb*_-reactive CD4^+^ T cells remained stable in mouse lungs and spleens, as did the number of Rel_*Mtb*_-reactive CD8^+^T cells. Therapeutic vaccination with *rel*_*Mtb*_ DNA vaccine enhanced the activity of isoniazid in Mtb-infected C57BL/6 mice and guinea pigs. When treatment with isoniazid was discontinued, mice immunized with the *rel*_*Mtb*_ DNA vaccine showed a lower mean lung bacterial burden at relapse compared to the control group. Our work shows that antitubercular treatment shapes the antigenic environment, and that therapeutic vaccination targeting the Mtb stringent response may represent a novel approach to enhance immunity against Mtb persisters, with the ultimate goal of shortening curative TB treatment.

## Introduction

Despite the high efficacy of the current 6-month “short-course” combination regimen, tuberculosis (TB) remains a global health emergency. Improper provision and supervision of treatment leads to excess morbidity and mortality, continued transmission, and emergence of drug resistance (1). The prolonged duration of curative TB treatment is believed to reflect the ability of a subpopulation of *Mycobacterium tuberculosis* (Mtb) bacilli to remain in a non-replicating persistent state in the infected host (2). These “persisters” exhibit reduced susceptibility to isoniazid (3), which inhibits mycolic acid synthesis in the cell wall (4, 5). This phenomenon is referred to as “antibiotic tolerance” (6), in which slowly dividing or non-dividing bacteria become less susceptible to killing by antibiotics targeting actively multiplying organisms.

One of the key bacterial pathways implicated in antibiotic tolerance is the stringent response, which is triggered by the rapid accumulation of the key regulatory molecules hyperphosphorylated guanosine [(p)ppGpp] and inorganic polyphosphate [poly(P)] in response to nutrient starvation and other stresses (7). Mtb has a dual-function enzyme, Rel_*Mtb*_, which is able to synthesize and hydrolyze (p)ppGpp (8, 9), as well as two polyphosphate kinases (PPK1, PPK2) and two exopolyphosphatases (PPX1, PPX2), which regulate intracellular poly(P) homeostasis (10–13). The mycobacterial stringent response appears to be a positive feedback loop, as poly(P) phosphorylates and activates the two-component system MprAB, which induces expression of *sigE* and *rel*_*Mtb*_ (14), leading to increased synthesis of (p)ppGpp, which inhibits the hydrolysis of poly(P) by the exopolyphosphatase, PPX2 (13). Deletion of *Rv2583/rel_*Mtb*_* results in profound bacterial phenotypes, including reduced Mtb survival under growth-limiting conditions *in vitro* (15), during chronic infection in mouse (16) and guinea pig (17) lungs, and in a mouse hypoxic granuloma model of latent TB infection (18). Rel_*Mtb*_ deficiency also results in increased susceptibility of Mtb to isoniazid during nutrient starvation and in mouse lungs (19).

Although the Mtb stringent response contributes to the formation of persisters and antibiotic tolerance, it remains to be determined whether this pathway can be targeted therapeutically during chronic Mtb infection. We have shown previously that DNA vaccination with four key stringent response genes (*rel_*Mtb*_, sigE, ppk2*, and *ppx1*) generated antigen-reactive CD4^+^ T-cell responses and vaccination prior to Mtb aerosol challenge augmented the tuberculocidal activity of isoniazid in mice (20). However, very limited data are available regarding the antigenic availability of stringent response factors during antitubercular treatment *in vivo*, as well as the optimal target for therapeutic DNA vaccination, which is an effective strategy for generating cellular and humoral immunity in various diseases (21). In the current study, we used the first-line anti-tubercular drug, isoniazid, as a tool to induce Mtb persistence *in vitro* and *in vivo* (22). We studied the expression of *rel*_*Mtb*_ in Mtb-infected macrophages treated with isoniazid. We then characterized the abundance of Rel_*Mtb*_-specific CD4^+^ T cells in the lungs and spleens of Mtb-infected C57BL/6 mice following daily oral treatment with human-equivalent doses of isoniazid. Finally, we investigated the efficacy of DNA vaccination targeting Rel_*Mtb*_ as an adjunctive therapy to isoniazid in two different animal models of chronic TB infection.

## Materials and Methods

### Bacteria and Growth Conditions

Wild-type Mtb H37Rv was grown in Middlebrook 7H9 broth (Difco, Sparks, MD) supplemented with 10% oleic acid-albumin-dextrose-catalase (OADC, Difco), 0.1% glycerol, and 0.05% Tween-80 at 37°C in a roller bottle (23).

### IC-21 Macrophages Infection and Real-Time PCR Analysis

The C57BL/6 macrophage cell line, IC-21 (ATCC, No. TIB-186), was grown in RPMI medium with 10% FBS and 1% penicillin/streptomycin. IC-21 cells were divided and plated in a multilayer culture flask (Millipore). At day 0, 10^6^ macrophages were infected with 5 × 10^6^ of logarithmically growing H37Rv bacilli. The cells were harvested 4 days after infection, RNA was extracted and qPCR analysis was performed, as described previously (20). The primers are listed in Table 1.

**TABLE 1:** Primers used for RT-qPCR.

**Primers used for qRT-PCR studies**	
*ppx1*	F: AGAGGACCCTAACGGCAAAT, R: TTTCCACCGCTTCTATCGAC
*relMtb*	F: GGGTGCTGGTGATAAAGGTG, R: AGGTCCTCCAACTCCCACTT
*ppk2*	F: CCTGGTACGTGGTGGAGTCT, R: TTGACCTTTGGCTTTTCCAC
*sigE*	F: AACTCGATTCGCTTGCTGAT, R: TGGTGAAACGTCAGCAGTTC
*rrs*	F: ATGCATGTCTTGTGGTGGAA, R: GTGCAATATTCCCCACTGCT
*esxA*	F: CGCAAGCTTATGACAGAGCAGCAGTGG,
	R: CGCGAATTCTGCGAACATCCCAGTGACGT
*sigA*	F: TCGAGGTGATCAACAAGCTG, R:TGGATTTCCAGCACCTTCTC

### Antigen Preparation

The previously generated *rel*_*Mtb*_ expression plasmid, pET15b[*rel*_*Mtb*_] (24), was used for expression and purification of recombinant Rel_*Mtb*_ protein. *Escherichia coli* BL21 (DE3) RP competent cells (Stratagene) were transformed with pET15b[*rel*_*Mtb*_]. Transformed bacteria were selected with ampicillin (100 μg/ml), and cloning was confirmed by DNA sequencing. Protein expression was performed using standard protocols and purification was performed using Ni-NTA Agarose (Qiagen). The Rel_*Mtb*_ protein (87 kDa) was purified from the cell lysate using a Ni-NTA resin column. The purity was confirmed by SDS-PAGE gel and immunoblot analyses (Supplementary Figure S1). The protein concentration was determined using a BCA protein assay with BSA as the standard (Thermo Fisher). Recombinant Rel_*Mtb*_ protein has been shown previously to retain (p)ppGpp synthesis and hydrolysis activities (24), and has been used as an antigen to measure Rel_*Mtb*_-specific T-cell responses *ex vivo* (20). Mtb peptides TB10.4 4 –11 (IMYNYPAM) and ESAT6 1–15 (MTEQQWNFAGIEAAA) (25) were commercially synthesized (Genescript), dissolved in DMSO and stored at 20°C until use. Mtb whole cell lysate (10 μg/ml, BEI) was used to measure Mtb-specific T cells during infection.

### DNA Vaccine

The plasmid pSectag2B encoding the full-length *rel*_*Mtb*_ gene was used as the *rel*_*Mtb*_ DNA vaccine (20). ESAT6 was PCR-amplified using forward (F) and reverse (R) primers (F: GCGAAGCTTGTGGCCGAGGACCAGCTCAC; R: CGCGGATCCGCGAACATCCCAGTGACGT) and cloned into pSectag2B using the restriction enzymes *Bam*HI and *Hin*dIII. Proper insertion was confirmed by sequencing. Each DNA vaccine was delivered as previously described (26, 27), and the original plasmid, pSectag2B, was used as a control. All procedures were performed according to protocols approved by the Johns Hopkins University Institutional Animal Care and Use Committee. Briefly, each plasmid was delivered by intramuscular injection into the quadriceps femoris muscle of mice (100 μg) or guinea pigs (500 μg) at the indicated time points.

### Harvest of Lung Cells and Splenocytes

At the indicated time points, the mice were sacrificed and peripheral blood and splenocytes were collected, as previously described (20, 27). At necropsy, the lungs were perfused with 1 ml of normal saline by direct injection into the right ventricle of the heart. A random section of the lungs was used for cytometry analysis. The tissue samples were incubated in RPMI medium (Gibco) containing collagenase D (1 mg/ml), DNase (0.25 mg/ml) and hyaluronidase type V (1 mg/ml) at 37°C for 1 h with intermittent agitation. The cells were then filtered through a 70-μm nylon filter mesh to remove undigested tissue fragments and washed with complete RPMI medium.

### Intracellular Cytokine Staining and Flow Cytometry Analysis

To detect antigen-reactive T-cell responses by IFN-γ intracellular staining, splenocytes or lung cells were stimulated individually with the purified recombinant proteins, Rel_*Mtb*_ (10 μg/ml), ESAT6 peptide (1 μg/ml) or TB10 peptide (1 μg/ml) for 24 h at 37°C before the addition of GolgiPlug (BD Pharmingen, San Diego, CA, United States). Protein and peptide concentrations were selected based on previous reports (20, 25). The cells were washed once with FACScan buffer and then stained with PE-conjugated monoclonal rat anti-mouse CD4 (BD Pharmingen) and/or APC-conjugated monoclonal rat anti-mouse CD8 (eBioscience). The cells were permeabilized using the Cytofix/Cytoperm kit (BD Pharmingen, San Diego, CA, United States). Intracellular IFN-γ was detected using FITC-conjugated rat anti-mouse IFN-γ (BD Pharmingen, San Diego, CA, United States). Flow cytometry was performed using FACSCalibur and the data were analyzed with FlowJo software.

### Aerosol Infection of Mice With Mtb and Therapeutic DNA Vaccination

Female C57BL/6 mice (6–8 week-old) were aerosol-infected with ∼100 bacilli of wild-type Mtb H37Rv. After 28 days of infection, the mice received isoniazid 10 mg/kg dissolved in 100 μl of distilled water by esophageal gavage once daily (5 days/week), and were randomized to receive DNA vaccine containing *rel*_*Mtb*_, *esat6*, or the empty vector (100 μg in water) by intramuscular injection (20). Five mice in each group were sacrificed on Days 28, 56, and 84 after aerosol infection. The lungs were homogenized in 5 ml of PBS using glass homogenizers. Serial tenfold dilutions of lung homogenates in PBS were plated on 7H11 selective agar (BD) at the indicated time points. Plates were incubated at 37°C and colony-forming units (CFU) were counted 4 weeks later (28).

### Aerosol Infection of Guinea Pigs With Mtb and Therapeutic DNA Vaccination

Female outbred Hartley guinea pigs (250 to 300 g) were purchased from Charles River Labs (Wilmington, MA, United States). Guinea pigs were aerosol-infected with Mtb H37Rv using a Madison chamber (University of Wisconsin, Madison, WI, United States) calibrated to deliver ∼2 log_10_ CFU to the lungs (29). After 28 days of infection, the guinea pigs received human-equivalent doses of isoniazid (60 mg/kg) dissolved in 300 μl of distilled water daily (5 days/week) by esophageal gavage (30). Concurrent with isoniazid treatment, the guinea pigs were randomized to receive either *rel*_*Mtb*_ DNA vaccine (500 μg) or empty vector (pSectag2B, 500 μg) in 500 μl of distilled water by intramuscular injection once weekly for 4 weeks. 28 days after treatment initiation, three guinea pigs in each group were sacrificed. Using a Kinematica Polytron Homogenizer with a 12-mm generator (Brinkman), the lungs were homogenized in 10 ml PBS within a BSL-III Glovebox Cabinet (Germfree). Serial tenfold dilutions of organ homogenates in PBS were plated on 7H11 selective agar (BD) at the indicated time points. The plates were incubated at 37°C and CFU were counted 4 weeks later (12).

### Enzyme-Linked Immunosorbent Assay

Antigen-specific antibody responses were measured by ELISA as described previously (31), with minor modifications in coating and sera incubation. The 96-well microplate was coated with purified Rel_*Mtb*_ (1 μg/ml) overnight. After blocking, serum from individually vaccinated guinea pigs was diluted 1:100 with PBS, added to the wells and incubated at room temperature for 2 h. HRP goat anti-guinea pig antibody (Abcam) was used for ELISA with guinea pig sera.

### Statistical Analysis

Data from at least three biological replicates were used to calculate means and standard error (SEM) for graphing purposes. To compare differences between experimental and control groups, statistical analyses employed the Mann-Whitney test for sample sizes less than 4 or unpaired student’s *t*-test for sample sizes >4, and a *p-*value of <0.05 was considered statistically significant. Pairwise comparisons of group mean values for log_10_ counts and flow cytometry data were made by using one-way analysis of variance and Bonferroni’s multiple comparison test posttest with GraphPad prism 7 (GraphPad, San Diego, CA, United States), and a *P*-value of <0.05 was considered significant.

## Results

### Isoniazid Treatment Shapes the Antigenic Environment and T-Cell Responses in Mtb-Infected Mice

It has been shown that antitubercular treatment decreases Mtb-specific T cells in mice (32, 33). C57BL/6 mice were aerosol-infected with Mtb H37Rv and 4 weeks later, were treated orally once daily with human-equivalent doses of isoniazid (10 mg/kg) (34) for a total of 4 weeks.

**FIGURE 1:**
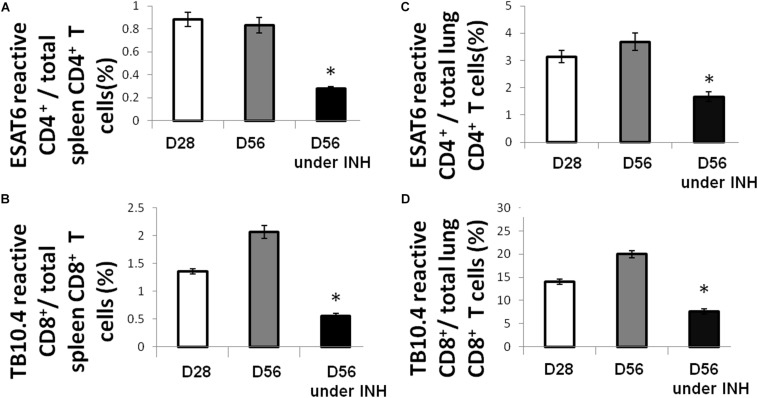
Isoniazid treatment decreases the proportions of immunodominant antigen-specific T cells. C57BL/6 mice were aerosol-infected with wild-type Mtb H37Rv and 4 weeks later, one group of mice received daily isoniazid (INH) treatment and the control group was untreated. After 4 weeks of treatment, ESAT6-reactive CD4^+^ **(A)** and TB10.4-reactive CD8^+^ **(B)** T cells in the spleen were measured by intracellular cytokine staining of IFNγ after stimulation with ESAT6 and TB10.4 peptides. Lung-derived ESAT6-reactive CD4^+^ **(C)** and TB10.4-reactive CD8^+^ **(D)** T cells were measured by intracellular cytokine staining of IFNγ after stimulation with ESAT6 and TB10.4 peptides. *N* = 3–4, *p < 0.05 compared to D56 without isoniazid treatment.

**FIGURE 2:**
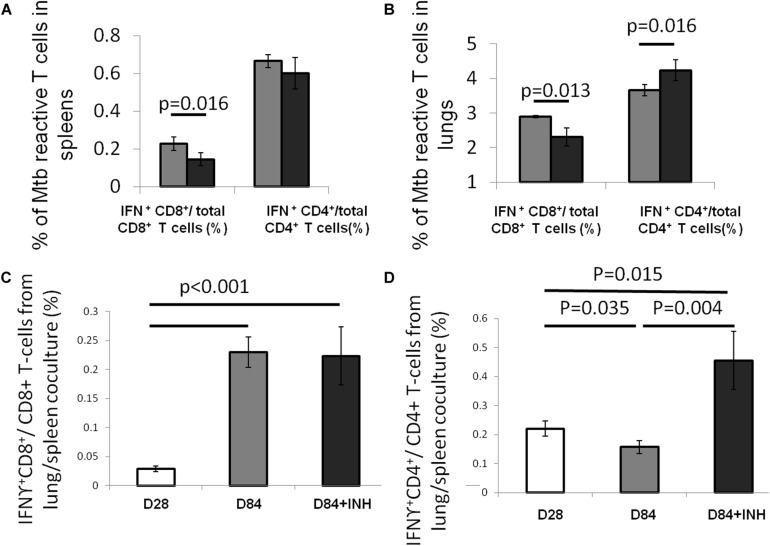
Isoniazid shapes antigenic environment and Mtb antigen-specific CD4 + T cells. C57BL/6 mice were aerosol-infected with wild-type Mtb H37Rv and 4 weeks later, one group of mice received daily isoniazid (INH) treatment (black bar) and the control group was untreated (gray bar). After 4 weeks of treatment, the splenocytes **(A)** and lung cells **(B)** were stimulated with Mtb lysate and Mtb-reactive CD4^+^ or CD8^+^ T cells were measured intracellular cytokine staining for IFNγ release. *N* = 5. To determine the antigenic environment during antibiotic treatment, the lung cells, including APCs presenting infection-generated antigens *in vivo*, from mice treated with isoniazid for 56 days and those from untreated mice were co-cultured with splenocytes from Mtb-infected C57BL/6 mice harvested 28 days after infection. IFNγ release from CD8^+^ **(C)** or CD4^+^ **(D)** T cells was measured by intracellular cytokine staining, *N* = 3–4.

Using an intracellular cytokine-releasing assay after *ex vivo* stimulation of splenocytes with peptides from the immunodominant Mtb antigens ESAT6 and TB10.4, we found that the amounts of ESAT6-reactive CD4^+^ and TB10.4-reactive CD8^+^ T cells that secreted IFNγ were significantly reduced in the lungs and spleens of Mtb-infected mice following isoniazid treatment (Figure 1). Next, we used Mtb lysate to stimulate splenocytes and lung-derived T cells. Intracellular cytokine staining revealed a significant reduction in IFNγ-releasing CD8^+^ T cells in the spleens, while IFNγ-releasing CD4^+^ T cells were increased in the lungs and unchanged in the spleens of Mtb-infected mice following 4 weeks of isoniazid treatment (Figures 2A,B).

In order to further characterize the effect of isoniazid treatment on Mtb antigen availability in the lungs of Mtb-infected mice, antigen presenting cells (APC) from the lungs of Mtb-infected/untreated mice (Day 28 or Day 84 after infection) or Mtb-infected/isoniazid-treated mice (Day 84 after infection/Day 56 of isoniazid treatment) were used to stimulate T cells from the spleens of a separate group of mice 28 days after aerosol infection with Mtb. The presented antigens of those APCs were naturally processed during Mtb infection *in vivo*. A significantly increased proportion of IFNγ-releasing CD4^+^ T cells was detected when splenocytes were co-cultured with lung cells from the 84-day infection with the treatment of isoniazid group relative to the untreated control group (Figures 2C,D). Our data have shown that the proportion of IFNγ-releasing CD4^+^ T cells following *ex vivo* stimulation with Mtb lysate increased in the lungs of isoniazid-treated, Mtb-infected mice, while the proportions of IFNγ-releasing CD4^+^ T and CD8^+^ T cells following *ex vivo* stimulation with ESAT6 peptide and TB10.4 peptide, respectively, decreased in the spleens and lungs of isoniazid-treated, Mtb-infected mice. Taken together, these findings indicate that isoniazid treatment shapes antigen presentation in the lungs of Mtb-infected mice.

### Isoniazid Induces Expression of Mtb Stringent Response Genes During Macrophage Infection

**FIGURE 3:**
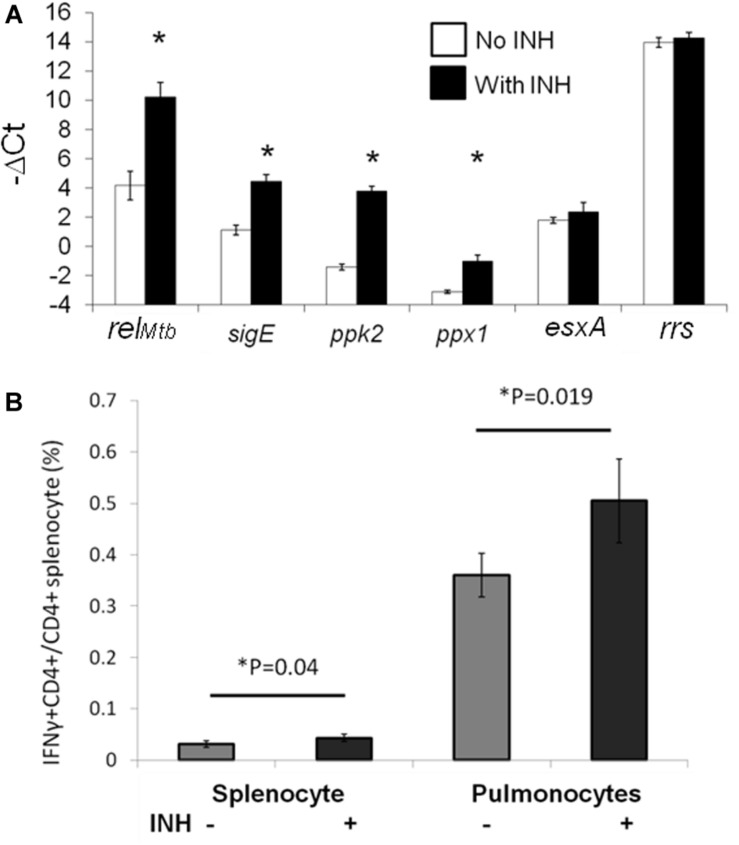
Rel_*Mtb*_ is upregulated during isoniazid treatment and presented in the lungs of Mtb-infected mice following isoniazid treatment. **(A)**. IC-21 macrophages were infected with Mtb H37Rv at an MOI 1:5 and were exposed to isoniazid (INH, 2 μg/ml) or left unexposed for 4 days. The intracellular bacilli were harvested to analysis expression of the stringent response genes by qPCR. The expression levels of each gene were normalized to that of the housekeeping gene, *sigA*. *N* = 3, *p < 0.05 compared to the untreated group. **(B)** C57BL/6 mice were aerosol-infected with wild-type Mtb H37Rv and 4 weeks later, one group of mice received daily isoniazid (INH) treatment and the control group was untreated. After 4 weeks of treatment, rel_*Mtb*_-reactive CD4^+^ T cells were measured by intracellular cytokine staining after isoniazid treatment in the spleens and lungs, *N* = 3–4.

We found previously that DNA vaccination targeting Mtb stringent response genes showed synergy with isoniazid against Mtb *in vivo*, despite having no demonstrable effect as a single treatment (20). To prioritize the four genes of our previously reported stringent-response vaccine (*rel_*Mtb*_, sigE, ppk2*, or *ppx1*) for further study, we used RT-PCR to study the expression of each gene during Mtb H37Rv infection of IC21 mouse macrophages, which were exposed or unexposed to isoniazid 2 μg/ml for 4 days (35). As shown in Figure 3A, *rel*_*Mtb*_ was the most highly induced gene following isoniazid exposure (*p* < 0.001). In contrast, expression of the Mtb genes encoding the immunodominant antigen ESAT6 and the housekeeping gene *rrs* did not change significantly after isoniazid exposure of Mtb-infected IC21 mouse macrophages.

### Rel_*Mtb*_ Antigen Continues to Be Presented During Isoniazid Treatment in Mice

In order to determine the significance of our findings and investigate Rel_*Mtb*_ antigen presentation by APCs during TB treatment in the mammalian host, we studied Rel_*Mtb*_-reactive CD4^+^ T-cell responses in the lungs of Mtb-infected mice treated with isoniazid. We harvested spleen-derived and lung-derived T cells from Mtb-infected and untreated C57BL/6 mice or infected mice treated with human-equivalent doses of isoniazid for 4 weeks. The harvested cells from the lungs or spleens were incubated *ex vivo* with recombinant Rel_*Mtb*_ (10 μg/ml) and Rel_*Mtb*_-reactive CD4^+^ T cells were detected using an intracellular cytokine-release assay. The proportion of Rel_*Mtb*_-reactive CD4^+^ T cells was modestly but significantly increased in the lungs and spleens of isoniazid-treated mice compared to untreated mice (Figure 3B). This is in contrast to the proportion of ESAT6-reactive lung T cells (Figure 1A), which decreased following isoniazid treatment compared to without treatment. These results suggest that the Mtb antigenic environment shifts during isoniazid treatment.

### Therapeutic DNA Vaccination Targeting Rel_*Mtb*_ Augments the Tuberculocidal Activity of Isoniazid in C57BL/6 Mice

**FIGURE 4:**
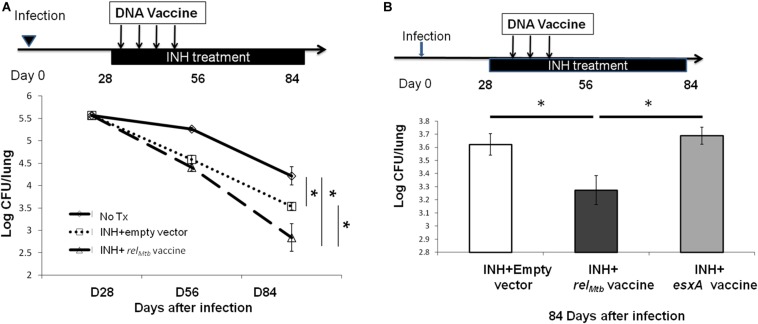
Vaccination targeting Rel_*Mtb*_ augments the bactericidal activity of isoniazid in Mtb-infected mice. **(A)** C57BL/6 mice were aerosol-infected with wild-type Mtb H37Rv and 28 days later, mice were treated with daily isoniazid (INH) and immunized with either the *rel*_*Mtb*_ DNA vaccine (100 μg) or the empty vector (100 μg) by intramuscular injection once weekly for 4 weeks. The lung CFUs were determined at the indicated time after treatment. *N* = 4–5. **(B)** C57BL/6 mice were aerosol-infected with wild-type Mtb H37Rv and 28 days later, mice were treated with daily isoniazid for 8 weeks. Concurrent with isoniazid treatment, separate groups of mice received 100 μg of the empty vector (control), *rel*_*Mtb*_ DNA vaccine, or *ESAT6* DNA vaccine by intramuscular injection once weekly for 3 weeks. The lung CFUs were determined 84 days after infection, *N* = 4–5. *p < 0.05.

Next, we tested whether DNA vaccination targeting the principal stringent response factor, Rel_*Mtb*_, could augment the bactericidal activity of isoniazid, as was observed for the tetravalent stringent-response vaccine (20). C57BL/6 mice were infected with the virulent Mtb strain H37Rv via aerosol. After 4 weeks of infection, all mice were treated orally with human-equivalent doses of isoniazid once daily (5 days/week) for 4 weeks. The mice were vaccinated either with *rel*_*Mtb*_ DNA vaccine (100 μg), or empty vector control (100 μg) once weekly for 4 weeks starting concurrently with isoniazid treatment. It should be noted that vaccine alone did not have a significant bactericidal effect in previous studies, so this group was not included here (20). After 4 weeks of treatment, adjunctive therapy with the monovalent *rel*_*Mtb*_ DNA vaccine lowered the mean lung bacillary burden by 0.695 log_10_ relative to isoniazid alone (*p* = 0.048, Figure 4A). To confirm the therapeutic specificity of the *rel*_*Mtb*_ DNA vaccine, we repeated the mouse study with a longer duration of treatment and added another group of mice, which received isoniazid plus DNA vaccine targeting ESAT6. After 8 weeks of treatment, the group receiving *rel*_*Mtb*_ DNA vaccine again showed a significant reduction in mean lung CFU compared to the empty vector group (*p* = 0.045, Figure 4B). In contrast, the mean lung bacillary burden of mice vaccinated with *esat6* DNA vaccine was not statistically different from that of control mice (Figure 4B). Based on these two independent studies, we conclude that immunization with *rel*_*Mtb*_ DNA vaccine potentiates the tuberculocidal activity of isoniazid in mice.

### *rel*_*Mtb*_ DNA Vaccination Reduces Mtb Regrowth Upon Discontinuation of Isoniazid Treatment

**FIGURE 5:**
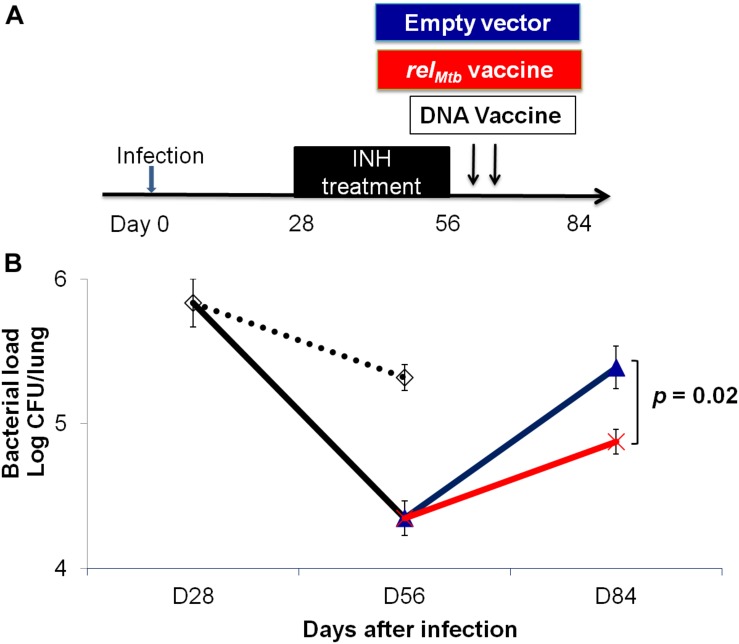
The *rel*_*Mtb*_ vaccine restricts Mtb regrowth following discontinuation of isoniazid treatment. **(A)** Experimental scheme. C57BL/6 mice were aerosol-infected with wild-type Mtb H37Rv and 4 weeks later, mice were either untreated (dots line) or treated with daily isoniazid (INH, black line) for 4 weeks, and treatment was discontinued. Immediately upon discontinuation of treatment, separate groups of mice received either 100 μg/ml empty vector DNA vaccine (blue line) or the *rel*_*Mtb*_ DNA vaccine (red line) by intramuscular injection once weekly for 2 weeks. **(B)** Lung bacillary burden (log_10_ CFU) at day 28, 56, and 84 after infection. *N* = 4–5.

To further study if *rel*_*Mtb*_ DNA vaccine can target persistent bacilli, C57BL/6 mice were aerosol-infected with Mtb and, 4 weeks later, were treated daily with isoniazid for 4 weeks. At the completion of isoniazid treatment, separate groups of mice received either *rel*_*Mtb*_ DNA vaccine (100 μg) or empty vector (100 μg) by intramuscular injection once weekly for 2 weeks (Figure 5A). Four weeks after cessation of isoniazid, the mean lung bacillary burden of mice receiving the *rel*_*Mtb*_ DNA was lower by 0.5 log_10_ compared to that of the empty vector vaccine group (*p* = 0.02, Figure 5B), suggesting that *rel*_*Mtb*_ DNA vaccine can restrict regrowth of Mtb after cessation of antibiotic therapy.

### *rel*_*Mtb*_ DNA Vaccine Enhances the Tuberculocidal Activity of Isoniazid in Guinea Pigs

**FIGURE 6:**
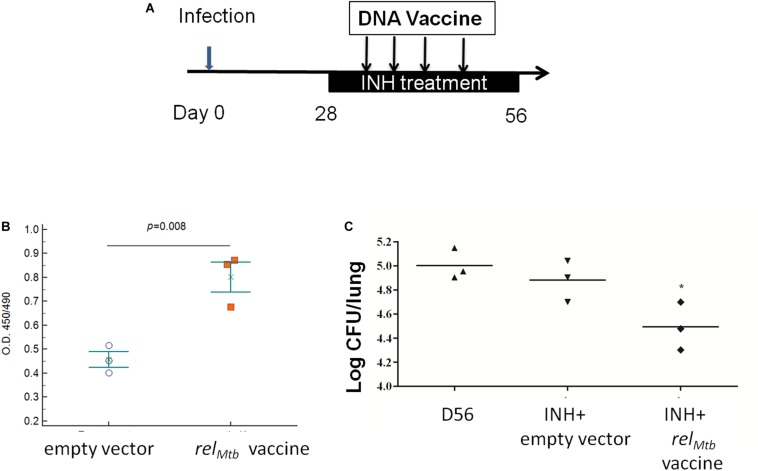
*rel*_*Mtb*_ DNA vaccine enhances the bactericidal activity of isoniazid in Mtb-infected guinea pigs. **(A)** Experimental scheme. Guinea pigs were aerosol-infected with wild-type Mtb H37Rv and, 4 weeks later, separate groups received oral isoniazid (INH) treatment (60 mg/kg) once daily in combination with either empty vector (control) or *rel*_*Mtb*_ DNA vaccine (500 μg) by intramuscular injection once weekly for a total of 4 weeks. **(B)** ELISA detection of anti-Rel_*Mtb*_ IgG antibody responses in the serum of groups receiving the empty vector or *rel*_*Mtb*_ DNA vaccine. **(C)** Mean lung bacillary burden (log_10_ CFU) at day 56 after infection (following 28 days of treatment), *p < 0.05.

To determine if the adjunctive antitubercular activity of the *rel*_*Mtb*_ DNA vaccine can be duplicated in different species, we infected guinea pigs with Mtb H37Rv via aerosol, and 4 weeks later, they were treated once daily with human-equivalent doses of isoniazid (60 mg/kg) (30) for a total of 28 days. Separate groups of guinea pigs were vaccinated either with *rel*_*Mtb*_ DNA vaccine (500 μg) or empty vector control (500 μg) once weekly for 4 weeks beginning with the initiation of isoniazid treatment (Figure 6A). To confirm the immunogenicity of the *rel*_*Mtb*_ DNA vaccine, we used ELISA to demonstrate Rel_*Mtb*_-specific IgG antibodies in the serum of vaccinated guinea pigs (Figures 6B), as we showed previously in mice receiving the stringent response DNA vaccine (20). At the time of treatment completion, the mean lung bacillary burden in the *rel*_*Mtb*_ DNA vaccine group was lower by ∼0.5 log_10_ relative to the that in the isoniazid alone control group (*p* = 0.05; Figure 6C), demonstrating that, as in mice, *rel*_*Mtb*_ DNA vaccine potentiates the antitubercular activity of isoniazid in guinea pigs.

## Discussion

Several potential reasons may explain the limited efficacy of immunodominant antigen-specific T cells in eliminating bacteria during chronic Mtb infection, including the reduced amount of immunodominant antigens presented, resulting in a reduction in the number of immunodominant antigen-specific CD4^+^ cells, as well as the continuous release of these antigens, leading to exhaustion of antigen-specific CD4^+^ T cells (36). Therefore, subdominant Mtb antigens, including those induced during antitubercular treatment, may represent better targets for therapeutic vaccines. In the current study, we show in the lungs of mice chronically infected with Mtb that oral treatment with the classical bactericidal drug isoniazid significantly maintains the proportion of CD4^+^ T cells reactive to the Mtb stringent response factor Rel_*Mtb*_, but those reactive to the Mtb immunodominant antigen ESAT6 were significantly decreased compared to the group receiving no isoniazid treatment. Furthermore, unlike the DNA vaccine targeting ESAT6, the DNA vaccine targeting Rel_*Mtb*_ potentiated the antitubercular activity of isoniazid in two different animal models of chronic TB infection.

Recently, there is significant interest in host-directed therapies to treat TB (37). Although BCG vaccination does not enhance the bactericidal activity of chemotherapy in the murine model (38), a DNA vaccine expressing heat shock protein 65 has been shown to synergize with conventional antitubercular drugs, further reducing the bacterial burden in the lungs of Mtb-infected mice or non-human primates (38, 39). A fragment whole cell lysate therapeutic vaccine, RUTI, has shown efficacy in generating protective immunity in pre-clinical studies (40, 41). However, the primary factors responsible for TB immunity during antibiotic treatment remain unknown. Previously, we have shown that a DNA vaccine targeting key Mtb stringent response factors generates IgG and antigen-specific CD4^+^ T cells, and increased the antitubercular activity of isoniazid when given as a therapeutic vaccine in Mtb-infected mice (20). The findings of the current study corroborate our previous findings and highlight the importance of immunity to Mtb stringent response factors during isoniazid treatment. One potential explanation for this phenomenon is that the population of persistent bacilli in untreated, chronically infected mice is relatively small (42), but exposure to isoniazid further drives the formation of drug-tolerant persisters (22, 30). In favor of this hypothesis, isoniazid exposure induces Mtb expression of Rel_*Mtb*_, which is required for persister formation (7, 19, 43, 44). Further studies are required to determine the potential utility of the *rel*_*Mtb*_ vaccine as an adjunctive therapeutic intervention in shortening the duration of treatment for active TB in mice. Additional studies are also needed to determine if *rel*_*Mtb*_ vaccine generates similar cellular responses in guinea pigs and to what extent the therapeutic efficacy of this vaccine is dependent on cellular vs. humoral immunity in mice and guinea pigs.

Our lung APC/spleen T-cell co-culture studies revealed that isoniazid treatment alters the antigenic environment in the lungs of Mtb-infected mice (Figures 2C,D). Interestingly, the proportion of ESAT6-reactive CD4^+^ and TB10.4-reactive CD8^+^ T cells were significantly decreased in the lungs (Figure 1), even though the proportion of Rel_*Mtb*_-reactive CD4^+^ T cells were modestly increased after isoniazid treatment (Figure 3). It is possible that reduced antigen presentation was associated with decreased antigen-specific T cells after treatment. Although this may be due to reduced antigen processing, the use of synthetic peptides to stimulate T cells *ex vivo* militates against this possibility, since antigen processing is not required. In a previous study (45), some dominant antigen-specific T cells declined in frequency, while others remained relatively stable in the peripheral blood compartment of patients with TB after 6 months of treatment. Isoniazid treatment appears to shape the antigens available in the lungs, decreasing the proportion of dominant antigens such as ESAT6 or TB10.4, resulting in differential T-cell responses. One potential explanation for these findings is that isoniazid treatment increases the amount of Rel_*Mtb*_ antigen available to lung APCs due to bacillary lysis. Alternatively, isoniazid treatment may have directly modified Mtb protein expression, leading to an altered antigenic environment. Besides the availability of antigen, antibiotic treatment may also affect the efficacy of antigen presentation by APCs, directly or indirectly. Furthermore, stimulation of T cells with purified protein from *E. coli.* may yield different responses compared to stimulation with peptides. Further studies are needed to determine the relevant epitope(s) for Rel_*Mtb*_ and to more directly compare changes in the proportions of ESAT6-reactive and Rel_*Mtb*_-reactive T cells following anti-tubercular treatment in mice. These studies will be important for excluding the possibility of differences in processing/presentation of different antigens, and can further guide the design of any future therapeutic vaccines.

The *rel*_*Mtb*_ DNA vaccine potentiated the killing activity of isoniazid against chronic TB infection C57BL/6 mice, which develop cellular lung granulomas lacking central necrosis, as well as in guinea pigs, which develop a potent delayed-type hypersensitivity response and more human-like granulomas with necrosis and tissue hypoxia (17). Interestingly, the adjunctive antitubercular activity of the *rel*_*Mtb*_ DNA vaccine was more pronounced in the guinea pig model, in which the defective survival phenotype of a *rel*_*Mtb*_-deficient Mtb mutant was significantly accelerated relative to the C57BL/6 mouse model (46, 47). These findings suggest that tissue necrosis is not a prerequisite for vaccine efficacy, but its presence may further enhance the activity of isoniazid. Further studies are needed to test the *rel*_*Mtb*_ DNA vaccine in other animal models with necrotic granulomas, including the C3Heb/FeJ mouse (48) and the non-human primate (49).

## Conclusion

In conclusion, we have shown that isoniazid treatment shapes the antigenic environment during chronic TB infection in mice, leading to altered antigen-specific CD4^+^ T-cell profiles in the lungs. In addition, this is the first study to use the Mtb stringent response protein Rel_*mtb*_ as a therapeutic vaccine target. Additional studies are needed to test the therapeutic efficacy of this vaccine in other animal models, and to characterize the immunological basis for its efficacy in mice and guinea pigs.

## Data Availability Statement

All datasets generated for this study are included in the article/Supplementary Material.

## Ethics Statement

The animal study was reviewed and approved by the Johns Hopkins University Institutional Animal Care and Use Committee.

## Author Contributions

Y-MC contributed to the design of the study, performance of the experiments, data interpretation, and writing of the manuscript. ND, VC, and MP contributed to the performance of the experiments. JG and RM contributed to the data interpretation and writing of the manuscript. C-FH contributed to the design of the study and data interpretation. PK contributed to the design of the study, data interpretation, and writing of the manuscript.

## Conflict of Interest

The authors declare that the research was conducted in the absence of any commercial or financial relationships that could be construed as a potential conflict of interest.
