# Supplementary material for: Antibiotic Treatment Shapes the Antigenic Environment During Chronic TB Infection, Offering Novel Targets for Therapeutic Vaccination
Source: Front Immunol. 2020 Apr 28;11:680. doi: 10.3389/fimmu.2020.00680 (PMC7198710; doi:10.3389/fimmu.2020.00680)
Supplement: Supplementary file 1 [file Image_1.pdf]

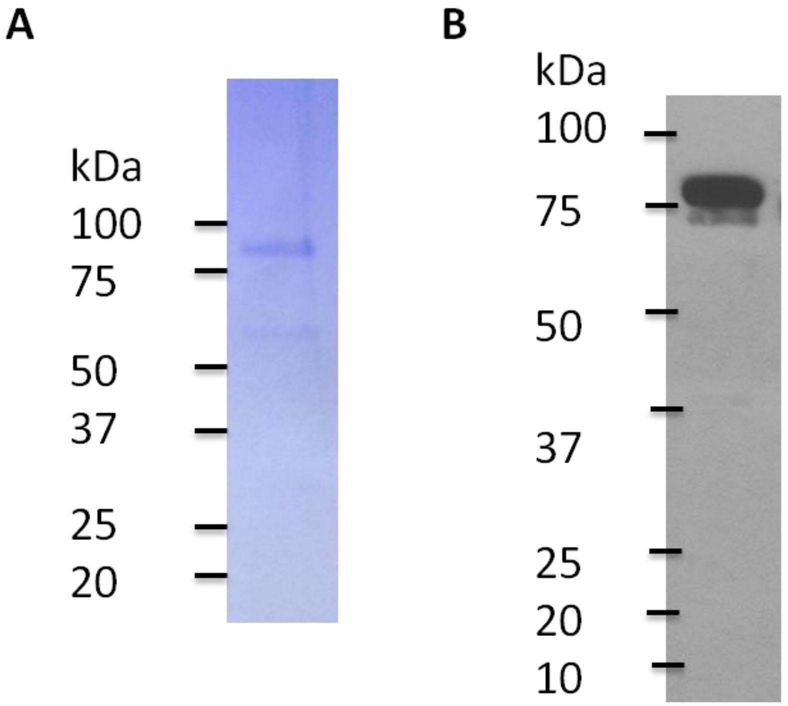

Supplement figure 1. The expression of Rel_{Mtb} protein from *E. coli.* A. SDS-PAGE analysis of purified recombinant Rel_{Mtb}. B. Immunoblot of purified recombinant Rel_{Mtb} probed with anti-Histag antibody.
